# Could 68Ga-PSMA PET/CT Evaluation Reduce the Number of Scheduled Prostate Biopsies in Men Enrolled in Active Surveillance Protocols?

**DOI:** 10.3390/jcm11123473

**Published:** 2022-06-16

**Authors:** Pietro Pepe, Marco Roscigno, Ludovica Pepe, Paolo Panella, Marinella Tamburo, Giulia Marletta, Francesco Savoca, Giuseppe Candiano, Sebastiano Cosentino, Massimo Ippolito, Andreas Tsirgiotis, Michele Pennisi

**Affiliations:** 1 Urology Unit, Cannizzaro Hospital, 95126 Catania, Italy; ludopepe97@gmail.com (L.P.); ppanella5@gmail.com (P.P.); francescosavoca@virgilio.it (F.S.); urocandia@gmail.com (G.C.); tsirgiotis.andreas@gmail.com (A.T.); michepennisi2@virgilio.it (M.P.); 2 Department of Surgery, UOC Urologia, ASST Papa Giovanni XXIII, 24127 Bergamo, Italy; roscigno.marco@gmail.com; 3 Radiotherapy Unit, Cannizzaro Hospital, 95126 Catania, Italy; marinella.tamburo@virgilio.it (M.T.); marlettagiulia1@gmail.com (G.M.); 4 Nuclear Medicine Unit, Cannizzaro Hospital, 95126 Catania, Italy; ianocose@hotmail.com (S.C.); massimo.ippolito@aoec.it (M.I.)

**Keywords:** prostate cancer, 68Ga-PSMA PET/CT, mpMRI, targeted prostate biopsy, active surveillance

## Abstract

Background: To evaluate the accuracy of 68Ga-prostate specific membrane antigen (PSMA) PET/CT in the diagnosis of clinically significant prostate cancer (csPCa) (Grade Group > 2) in men enrolled in Active Surveillance (AS) protocol. Methods: From May 2013 to May 2021, 173 men with very low-risk PCa were enrolled in an AS protocol study. During the follow-up, 38/173 (22%) men were upgraded and 8/173 (4.6%) decided to leave the AS protocol. After four years from confirmatory biopsy (range: 48–52 months), 30/127 (23.6%) consecutive patients were submitted to mpMRI and 68Ga-PSMA PET/CT scan before scheduled repeated biopsy. All the mpMRI (PI-RADS > 3) and 68Ga-PET/TC standardised uptake value (SUVmax) > 5 g/mL index lesions underwent targeted cores (mpMRI-TPBx and PSMA-TPBx) combined with transperineal saturation prostate biopsy (SPBx: median 20 cores). Results: mpMRI and 68Ga-PSMA PET/CT showed 14/30 (46.6%) and 6/30 (20%) lesions suspicious for PCa. In 2/30 (6.6%) men, a csPCa was found; 68Ga-PSMA-TPBx vs. mpMRI-TPBx vs. SPBx diagnosed 1/2 (50%) vs. 1/2 (50%) vs. 2/2 (100%) csPCa, respectively. In detail, mpMRI and 68Ga-PSMA PET/TC demonstrated 13/30 (43.3%) vs. 5/30 (16.7%) false positive and 1 (50%) vs. 1 (50%) false negative results. Conclusion: 68Ga-PSMA PET/CT did not improve the detection for csPCa of SPBx but would have spared 24/30 (80%) scheduled biopsies showing a lower false positive rate in comparison with mpMRI (20% vs. 43.3%) and a negative predictive value of 85.7% vs. 57.1%, respectively.

## 1. Introduction

In the last decade, active surveillance (AS) has become an alternative to radical treatment of low-/very low-risk prostate cancer (PCa), focusing on prevention of overtreatment (50% of the cases in screening studies) [1,2,3]. Multiparametric magnetic resonance imaging (mpMRI) has demonstrated good accuracy in diagnosing clinically significant PCa (csPCa), particularly if the cancer is located in the anterior prostate [4]; therefore, mpMRI is now strongly recommended in AS follow-up [5]. However, the time of confirmatory biopsy has been established within one year from initial diagnosis [6], and there are no data regarding the number of systematic needle cores and the best imaging procedure to use for omitting or postponing scheduled repeated biopsies.

Recently, prostate-specific membrane antigen (PSMA) inhibitors conjugated with the radionuclides 68Ga and 18F-fluoride have been well-explored and successfully translated for the clinical diagnosis of PCa [7,8]. Moreover, tumour uptake, which represents PSMA expression, results were highly correlated with the Gleason score of the primary prostatic tumour [9]. However, in a limited number of studies focused on the primary prostatic lesion, 68Ga-PSMA positron emission tomography/computed tomography (PET/CT) has been shown to be sensitive for the detection of primary prostatic lesions and regional lymphadenopathy [10,11,12]. Recently, Raveethiran et al. suggested that the addition of a diagnostic 68Ga-PSMA PET/CT to mpMRI can improve the detection of significant prostate cancer and improve the ability to identify men suitable for active surveillance [13].

The aim of this study was to prospectively evaluate the diagnostic accuracy of 68Ga-PSMA PET/CT and mpMRI in the diagnosis of csPCa (Grade Group > 2) [14] in men enrolled in AS protocol.

## 2. Materials and Methods

From May 2013 to May 2021, 173 men aged between 52 and 73 (median age 63) with very low-risk PCa were enrolled in an AS protocol study. After institutional review board and ethical committee approval were granted, informed consents were obtained from all participants included in the study. Presence of the following criteria defined eligibility: life expectancy greater than 10 years, clinical stage T1C, PSA below 10 ng/mL, PSA density (PSA-D) < 0.20, <2 unilateral positive biopsy cores, Gleason score 6/International Society of Urologic Pathology (ISUP) Grade Groups (GG) 1 [14] and maximum core percentage of cancer (GPC) < 50% (3). All the patients underwent confirmatory biopsy six months after the PCa diagnosis and mpMRI evaluation. During the follow-up, 38/173 (22%) men were upgraded and 8/173 (4.6%) men autonomously decided to leave the AS protocol. After four years from confirmatory biopsy (range: 48–52 months), also in the presence of stable clinical parameters, the last 30/127 (23.6%) consecutive patients were submitted to mpMRI and 68Ga-PET/CT imaging examinations before scheduled repeated biopsy.

All mpMRI examinations were performed using a 3.0 Tesla scanner (ACHIEVA 3T; Philips Healthcare, Best, The Netherlands) equipped with 16 surface channel phased-array coils placed around the pelvic area with the patient in the supine position; multi-planar turbo spin-echo T2-weighted (T2W), axial diffusion weighted imaging (DWI) and axial dynamic contrast enhanced (DCE) were performed for each patient. The mpMRI lesions characterised by Prostate Imaging Reporting and Data System (PI-RADS) version 2 (4) scores > 3 were considered suspicious for cancer; two radiologists blinded to pre-imaging clinical parameters evaluated the mpMRI data separately and independently; moreover, one urologist with more than 25 years of experience performed the biopsy procedure [6]. The data were collected following the Screening Tool to Alert to Right Treatment (START) criteria [15].

**Figure 1:**
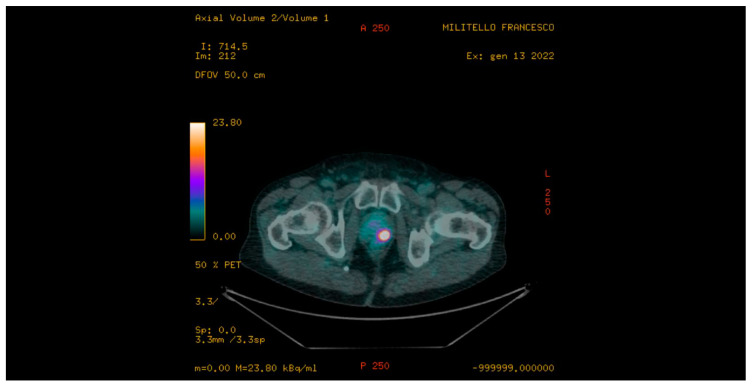
68Ga-prostate-specific membrane antigen (PSMA) PET/CT: presence of high suspicious area of prostate cancer in the left lobe of prostate gland (axial valuation) with a standardised uptake value (SUVmax) equal to 19.8 g/mL. Targeted biopsy demonstrated the presence of a Grade Group 2 prostate cancer.

PET/CT imaging was performed using a CT-integrated PET scanner (Biograph 6; Siemens, Knoxville, TN, USA). 68Ga-PSMA was prepared with a fully automated radiopharmaceutical synthesis device based on a modular concept (Eckert & Ziegler Eurotope, Berlin, Germany). 68Ga-PSMA-11 was given to patients via an intravenous bolus (mean, 144 ± 12 MBq; range, 122–188 MBq), and the PET acquisition was started at a mean of 58 ± 12 min (range, 50–81 min) afterward. Scans were acquired in 3-dimensional mode with an acquisition time of 3 min per bed position. Emission data were corrected for randoms, dead time, scatter and attenuation and were reconstructed iteratively using ordered-subsets expectation maximisation (4 iterations, 8 subsets) followed by a post-reconstruction smoothing Gaussian filter (5 mm in full width at half maximum). For attenuation correction, a low-dose unenhanced CT scan was performed from the skull base to the middle of the thigh. Images were processed to obtain PET, CT, and PET-CT fusion sections in the axial, coronal, and sagittal planes with a thickness of approximately 0.5 cm by two experienced nuclear medicine specialists, who were blinded to the clinical data. The location of focal uptake on 68Ga-PSMA PET/CT (Figure 1), three-dimensional size, and standardised uptake value (SUVmax) values were reported on a per-lesion basis with a sexstant scheme (apex, midgland and base, each split into left and right) [7].

All the mpMRI (PI-RADS score > 3) and 68Ga-PET/TC index lesions (SUVmax > 5 g/mL) [15] underwent targeted cores (mpMRI-TPBx and PSMA-TPBx: four cores) combined with saturation prostate biopsy (SPBx: median 20 cores; range 18–22). The procedure was performed transperineally using a tru-cut 18-gauge needle (Bard; Covington, GA, USA) under sedation and antibiotic prophylaxis [16]. The prostate targeted cores were done using an Hitachi 70 Arietta ecograph, Chiba, Japan) supplied by a bi-planar trans-rectal probe [17] performing a free-hand cognitive approach.

## 3. Results

The clinical parameters of the 30 men enrolled in the active surveillance protocol are listed in Table 1. No selection criteria were used for patients submitted to PET-PSMA evaluation, and no significant differences in terms of clinical parameters were found between these patients and the entire active surveillance group.

Multiparametric MRI and 68Ga-PSMA showed 14/30 (46.6%) and 6/30 (20%) lesions suspicious for PCa those were submitted to targeted cores combined with SPBx. In detail, mpMRI PI-RADS score resulted < 2 vs. 3 vs. 4 in 16 (53.3%) vs. 12 (40%) vs. 2 (6.7%) men. The average intraprostatic SUVmax and tumor dimension was 4.8 g/mL (range: 3.2–19.8) and 7.3 mm (range 4–12 mm), respectively; only 6/30 (20%) men had a SUVmax > 5 g/mL (range: 5.1–19.8 g/mL), moreover, 68Ga-PSMA PET/TC showed two suspicious areas in correspondence of iliac ala and spinal cord; were shown to be negative for metastases in targeted MRI for bone evaluation. In 2/30 (6.6%) men, a csPCa (GG2) was found: both patients had a GPC equal to 20% with a number of positive cores equal to 3 and 4, respectively. PSA density was 0.15 and 0.11.

68Ga-PSMA-TPBx vs. mpMRI-TPBx vs. SPBx diagnosed 1/2 (50%) vs. 1/2 (50%) vs. 2/2 (100%) csPCa, respectively. In detail, PET/CT PSMA and mpMRI missed the diagnosis of csPCa in two different patients: one patient had a PI-RADS score of 2 and SUVmax of 6.8 g/mL; the man not detected by PSMA PET had a PI-RADS of score 3 at moMRI and SUVmax equal to 4.5 g/mL at 68Ga-PET/TC. In detail, mpMRI and 68Ga-PSMA PET/TC demonstrated 13/30 (43.3%) vs. 5/30 (16.7%) false positive and 1 (50%) vs. 1 (50%) false negative results. In addition, mpMRI and 68Ga-PSMA PET/TC showed a negative predictive value (NPV) in the diagnosis of csPCa equal to 57.1 and 85.7%, respectively.

## 4. Discussion

The estimated risk-free treatment at 5, 10 and 15 years in men enrolled in AS with GG1 PCa is equal to 76, 64 and 58% [1]; in the last years, many studies have been reported suggesting the best protocol of follow up to reduce the number on scheduled prostate biopsies [1,2]. In this respect, although mpMRI is strongly recommended in the revaluation of men in AS [2,5], scheduled systematic repeated prostate biopsies are still recommended in addition to targeted mpMRI/TRUS fusion biopsy to reduce the false negative rate for csPCa of mpMRI equal to 20% of the cases [17]. At the same time, the number of cores performed at initial repeat evaluation is directly correlated with a lower risk of reclassification [6] during the follow-up, allowing to postpone scheduled repeated prostate biopsy in favour of clinical findings (i.e., PSA density, risk calculator) [18] and imaging revaluation (mpMRI) [5,16,19].

Recently, 68Ga-PSMA-PET/CT has been suggested to improve the clinical stadiation of high-risk PCa and disease recurrence [7]; at the same time, PSMA PET/CT has been proposed for the diagnosis of primary intraprostatic cancer [15]. The presence of focal uptake on PSMA-PET/CT, the standardised uptake value (SUVmax) and the maximal dimensions of PET-avid lesions have been correlated with the presence of csPCa [20,21]. There is a range of proposed cutoffs to detect csPCa from SUVmax 3.15 to up SUVmax 9.1 [22,23]; the concordance between preoperative PSMA PET/TC evaluation (SUVmax, dimension of the lesion), and definitive prostate specimen ranges from 81.2% (24) to 96% [24,25,26,27,28]. Moreover, PSMA PET/MRI seems to reduce the false positive rate of PET/CT (about 8% of cases) [26].

To our knowledge, this is the first study that prospectively evaluated the role of 68Ga- PSMA PET/CT in men enrolled in prostate cancer AS protocols [29]. In our series, 68Ga-PSMA-TPBx vs. mpMRI-TPBx vs. SPBx diagnosed 1/2 (50%) vs. 1/2 (50%) vs. 2/2 (100%) csPCa, respectively. In detail, mpMRI and 68Ga-PSMA PET/TC demonstrated 13/30 (43.3%) vs. 5/30 (16.7%) false positive and 1 (50%) vs. 1 (50%) false negative results. In addition, mpMRI and 68Ga-PSMA PET/TC showed an NPV in in the diagnosis of csPCa equal to 57.1 and 85.7%, respectively.

Diagnostic imaging should not replace scheduled prostate biopsy but is mandatory to detect targeted lesions suspicious for csPCa. Several biochemical parameters, such as thymidine kinase I, mindin or PHI, could be helpful in decrease the ratio of scheduled biopsy. We have no data about these parameters; however, we evaluated our patients according to PSA density, as suggested by latest EAU guidelines.

Among our results, some considerations should be made. First, the number of patients evaluated was low; secondly, the results should be evaluated in the entire prostate specimen and not in biopsy histology. A more detailed histological evaluation of patients who underwent biopsy upstaging would be of interest, for example by adding supplementary staining for PSMA on the biopsy samples. However, this type of staining is not routinely performed at our institution. Third, the low rate of reclassification (6.6% of the cases) could be explained because the patients previously underwent SPBx plus mpMRI evaluation before confirmatory biopsy. Four, 68Ga-PSMA PET/TC evaluation could be proposed in men with negative mpMRI or in the presence of claustrophobia or cardiac pacemaker. Finally, a 68Ga-PSMA PET/TC fusion platform would have increased the accuracy of targeted prostate biopsy.

In conclusion, 68PSMA PET/CT did not improve the detection for csPCa of SPBx (1 false negative result equal to 50% of the cases); at the same time, 68Ga-PSMA PET/CT would have spared 24/30 (80%) scheduled biopsies showing a lower false positive rate in comparison with mpMRI (20% vs. 43.3%) and a better NPV (85.7 vs. 57.1%).

## Figures and Tables

**Table 1:** Clinical parameters of 30 men with low-risk prostate cancer (PCa) submitted to scheduled biopsy.

Clinical and Biopsy Findings	GG1 30 Patients
**Median PSA** **(range: 4.5–122 ng/mL)**	4.6
**Median PSA density** **(range: 0.10–0.21)**	0.15
**Median GPC (range: 10–50%)**	40%
**Median number of positive cores** **Percentage of positive cores**	2 9%
**mpMRI** **PI-RADS score > 3**	13 (43.3%)
**68Ga-PSMA PET/CT** **suspicious for PCa**	6 (20%)

**Legend:** GG: International Society of Urological Pathology Grade Group; mpMRI: multiparametric magnetic resonance imaging; PSA: prostate-specific antigen; GPC: greatest percentage of cancer; PSMA: Prostate specific membrane antigen; PI-RADS: prostate imaging reporting and data system; PET/TC: positron emission tomography/computed tomography.

## Data Availability

Data are available at the institutional prostate cancer database of the Urology Unit, Cannizzaro Hospital, Catania.
